# Time-Resolved Study of Nanoparticle Induced Apoptosis Using Microfabricated Single Cell Arrays

**DOI:** 10.3390/microarrays5020008

**Published:** 2016-04-15

**Authors:** Peter J. F. Röttgermann, Kenneth A. Dawson, Joachim O. Rädler

**Affiliations:** 1 Faculty of Physics and Center for NanoSciene (CeNS), Ludwig-Maximilians-Universität, Geschwister-Scholl-Platz 1, 80539 Munich, Germany; peter.roettgermann@physik.lmu.de; 2 Centre for BioNano Interactions, School of Chemistry and Chemical Biology, University College Dublin, Belfield, Dublin 4, Ireland; Kenneth.A.Dawson@cbni.ucd.ie

**Keywords:** single cell array, cytotoxicity, nanoparticle, dose-response, correlation studies, micropatterning

## Abstract

Cell fate decisions like apoptosis are heterogeneously implemented within a cell population and, consequently, the population response is recognized as sum of many individual dynamic events. Here, we report on the use of micro-patterned single-cell arrays for real-time tracking of nanoparticle-induced (NP) cell death in sets of thousands of cells in parallel. Annexin (pSIVA) and propidium iodide (PI), two fluorescent indicators of apoptosis, are simultaneously monitored after exposure to functionalized polystyrene ($\mathrm{PS}-{\mathrm{NH}}_{2}$) nanobeads as a model system. We find that the distribution of Annexin onset times shifts to later times and broadens as a function of decreasing NP dose. We discuss the mean time-to-death as a function of dose, and show how the ${\mathrm{EC}}_{50}$ value depends both on dose and time of measurement. In addition, the correlations between the early and late apoptotic markers indicate a systematic shift from apoptotic towards necrotic cell death during the course of the experiment. Thus, our work demonstrates the potential of array-based single cell cytometry for kinetic analysis of signaling cascades in a high-throughput format.

## 1. Introduction

The interactions of nanoparticles (NPs) with cells remain poorly understood and this has raised concerns about potential cytotoxicity and environmental risks [1,2]. In recent years, many *in-vitro* and *in-vivo* studies have probed the safety and biocompatibility of NPs. Evidence for cytotoxicity was found in particular cases of NPs, depending on the cell line and test conditions used [3,4,5,6]. The majority of studies uses population-based toxicity assays, such as colorimetric assays for cell viability [7,8] and DNA fragmentation assays [9], or techniques with single-cell sensitivity, such as flow cytometry [10,11], image cytometry [12], or fluorescence microscopy [3], but data are taken at limited number of specific time points. It has recently been noted that cell-to-cell variations, which are averaged out in populations measurements but are revealed in single cell analysis, have non-genetic origins and provide important information on noise in apoptosis regulating circuitry [13,14]. Naturally occurring fluctuations in the levels of regulatory proteins can lead to ”fractional killing” and subpopulations of very sensitive or robust cells [14,15,16]. Moreover, time-lapse microscopy allows for fully time-resolved studies, in which every cell is tracked over time via brightfield and fluorescence microscopy [17,18,19]. These studies can directly assess the heterogeneous dynamic response of individual cells.

It has become clear that, in NP toxicity studies, the precise experimental conditions have a crucial bearing on the results, and great care is required in the preparation and administration of NPs. Depending on the biological media chosen, NPs may be coated with a protein corona that further facilitates their entry into cells and determines their effect on cells [20,21]. However, we still know little about the biochemical pathways that are affected by NPs and how NPs eventually induce cell death. In order to understand the internal signaling processes and discriminate between various pathways that lead to cell death, it is crucial to measure cellular responses to NPs at the single-cell level using quantitative readouts. Typical cell death markers used in microscopy are Annexin V and propidium iodide (PI). The Annexin V-based marker pSIVA shows increased fluorescence when bound to phosphatidylserine (PhS), and hence indicates the externalization of plasma-membrane PhS induced by activation of the caspase-dependent pathway. The impermeable dye PI stains the nucleus only when the integrity of the cell membrane is lost, and this can be related to the late stage of apoptosis, the so-called secondary necrosis [22,23]. The use of cells captured on microfluidic- [24] or micro-patterned cell arrays offers a route towards high-throughput analysis. We recently introduced micro-patterned substrates for time-resolved measurements on regularly arrayed cells, and showed that cells self-organize onto fibronectin-coated sites surrounded by boundaries passivated by treatment with poly-l-lysine- polyethylene glycol [25,26].

Here, we perform NP toxicity studies on single cell arrays which yield time-resolved data at single-cell resolution. For a first proof of concept, we choose hepato carcinoma derived HuH7 liver cells exposed to $\mathrm{PS}-{\mathrm{NH}}_{2}$ NPs, because liver cells are relevant in bio-accumulation and frequently used in toxicity studies. The timing of the onset of activation of two fluorescent markers — pSIVA, indicating the early apoptotic events and PI, the late stage of apoptosis or necrosis — was measured and the corresponding distribution function was analyzed as a function of dose. We show that the dynamics of NP-induced apoptosis is dose dependent, and relate the time-to-death value $\tau_{50}$ to the effective ${\mathrm{EC}}_{50}$ value. Furthermore, we find that the timing and signal intensities of the different apoptosis events are correlated.

## 2. Results

### 2.1. Highly Parallel Assessment of the Kinetics of Cell Death

**Figure 1:**
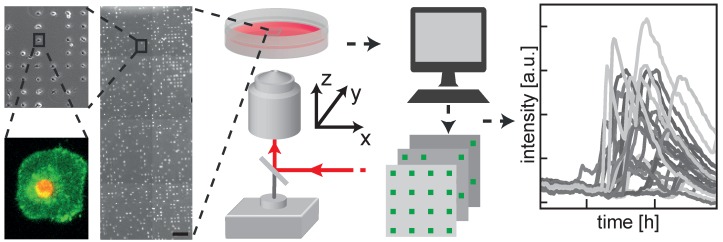
Protein-coated arrays are generated following plasma-induced patterning of μ-dishes. Cells distribute themselves on the patterned sites of an array after seeding. They are then exposed to NPs and monitored for activation of fluorescent markers of cell death for up to 72 h using an automated fluorescence microscope. The ease of automated image processing on the cell lattice enables high-throughput analysis of the kinetics of single-cell fluorescence. Scale bar: 500 μm (close-ups 5× and 25× magnified, respectively).

Figure 1 shows the workflow for data acquisition in time-resolved single-cell measurements. Microstructured surfaces for the preparation of single cell arrays are fabricated by selective plasma-induced patterning on either dishes or 8 well slides. Cells are seeded onto the microstructures and are left for five hours to self-arrange and adhere to the fibronectin adhesion sites. Cells are then exposed to different doses (0.1 to 100 μg · mL${}^{-1}$) of $\mathrm{PS}-{\mathrm{NH}}_{2}$ NPs, and incubated together with the fluorescent markers pSIVA-IANBD (an Annexin B12 derivative) and PI. No further washing step is applied. NPs were obtained from the QualityNano project and were characterized by transmission electron microscopy (see Supplementary 1, Figure S1). The patterned cell samples are then scanned at 10-min intervals under physiological conditions for up to 48 h, using an automated microscope. Typically, 500 single cell traces of fluorescence intensities are extracted from image stacks using grid-based read-out software.

**Figure 2:**
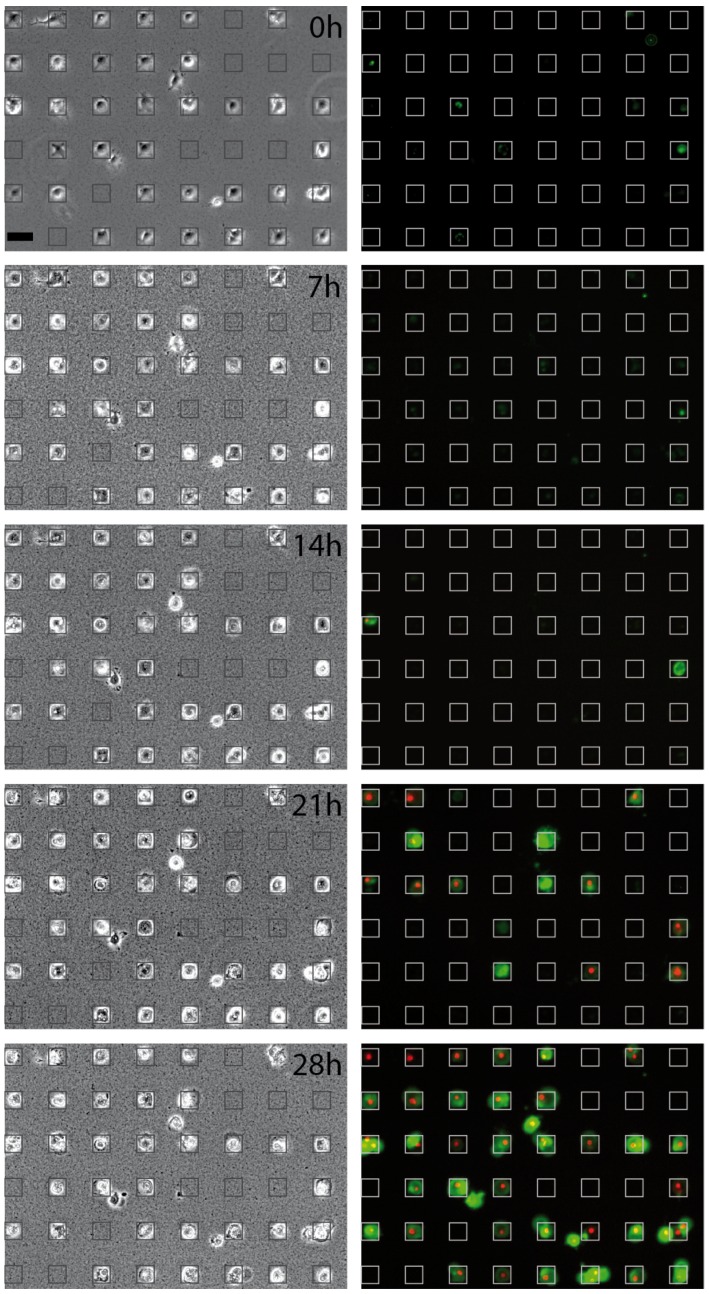
Time series of apoptotic events induced by $\mathrm{PS}-{\mathrm{NH}}_{2}$-NPs monitored by brightfield illumination and fluorescence imaging at 0, 7, 14, 21 and 28 h after seeding. The graininess seen in the brightfield images is attributed to sedimentation of nanoparticle (NP) aggregates. In the fluorescence images, green staining (pSIVA-IANBD) indicates exposure of phosphatidylserine (PhS) on the outer leaf of the plasma membrane bilayer, the red nuclear staining (PI) indicates subsequent loss of plasma membrane integrity. The heterogeneity in the times of onset of apoptosis can be clearly seen. Square lattices are drawn for better visualization. Scale bar: 50 μm.

Figure 2 shows brightfield (left) and fluorescence (right) microscopy images taken at selected time-points (0, 7, 14, 21 and 28 h, see movie in Supplementary 2). Green fluorescing cells indicate the binding of pSIVA to the cell surface at an early stage of apoptosis. During the the first 14 h, few cells show green fluorescence, and fluorescence emission begins in earnest only after 20 h. A similar pattern is observed for the red PI signal, albeit with a somewhat later onset (see below). In the time series and in Movie S2, the subsequent onset times of the green and red signal is clearly visible. The fading of the green signal towards the end of the measurement (28 h) is due to bleaching of the IANBD fluorophore.

**Figure 3:**
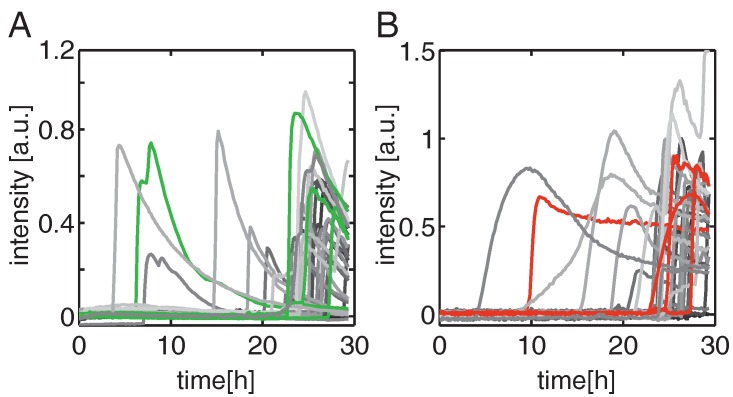
Representative time traces of fluorescent signals monitored in the polarity Sensor for Viability and Apoptosis (pSIVA) (**A**) and propidium iodide (PI) (**B**) channels. A few typical traces are highlighted in green and red, respectively. The first apoptotic events are observed after 3 h whereas the majority of induced cell deaths occur after 20 h. The decrease in the pSIVA signal at late times can be attributed to bleaching of the fluorophores. Fluorescent signals are background corrected.

Fluorescence intensities integrated over the adhesion sites (indicated as squares in Figure 2) for each single cell can be extracted from the image stacks, and Figure 3 shows the time courses of single-cell intensities as a function of the NP exposure time. The plasma membrane transition of PhS in individual cells is readily detectable from the time course of the pSIVA signal (Figure 3A).

Loss of plasma membrane integrity is indicated by a sharp increase of the PI signal (Figure 3B). The times of onset of these changes in individual cells can be clearly seen for pSIVA as well as for PI. For an NP dose of 10 μg · mL${}^{-1}$, the first apoptotic events are seen after 3 h and most cell deaths occur about 20 h after administration of NPs. Although not discernible in Figure 3B, in any given cell the PI signal appears about 70 min on average after the onset of the pSIVA signal (see below). The pSIVA time courses typically reveal a sudden, sharp increase followed by an exponential decrease. The latter behavior is attributable to the bleaching of the fluorophores. Some pSIVA traces show a second peak after the onset. Morphological changes such as formation of apoptotic bodies can lead to an increase in area and hence cause a higher mean fluorescence signal. In contrast, the decrease in the PI signal after the onset of cell death is related to non-specific staining of RNA, which lowers the intensity of PI fluorescence [27]. Most notably the peak intensities of both pSIVA and PI time courses vary quite considerably. The peak intensity distribution are plotted in the Supplementary Figure S3A, and we will discuss this cell-to-cell variability later on. In the following we focus on the onset-times of the pSIVA and PI signals. In our automated data analysis these values were determined as the times at which the signal first exceeds a level equivalent to 25% of the maximum background intensity. Cells which were dead from the beginning (less than 5%) are excluded from analysis. Also cell sites showing multiple occupation were excluded. Multiple occupation occurs either by the attachment of two cells on the same site or by cell division. The exclusion of dividing cells could lead to a bias in the cell death population as cells in a late cell cycle are neglected. However, the percentage of dividing cells over 40 hours is relatively low, about 15% in the case of cell arrays without exposure to NPs. Addition of NPs even decreases the ratio to 10% for a dose of 0.1 μg · mL${}^{-1}$ and below 1% for higher doses. This decreased cell division rate is due to cell-cycle arrest [28]. Hence, as the fraction of dividing cells is low, the restriction of our analysis to pure single cell events should not adversely bias the assessment of cell death timing.

### 2.2. Time Dependent Dose Response Function

**Figure 4:**
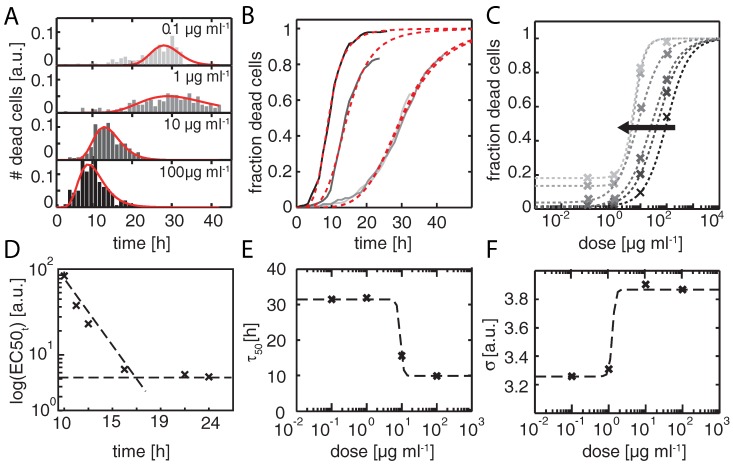
(**A**) Normalized frequency distributions of times of cell death (onset times of the PI signal) are plotted against time for the indicated NP doses. Note that the distributions shift to earlier time points and get narrower with increasing NP dose. The distributions are fitted to log-normal functions (red curve); (**B**) The cumulative fraction of dead cells is plotted against time for NP doses of 0.1 μg · mL${}^{-1}$ (light gray), 1 μg · mL${}^{-1}$ (medium gray), 10 μg · mL${}^{-1}$ (dark gray), and 100 μg · mL${}^{-1}$ (black). At the lowest dose 20% of the cells die within 24 h, whereas at the highest dose all cells (100%) are dead by this point. The cumulative distributions are fitted to log-normal functions (red curve); (**C**) Standard dose-response curves with fraction of dead cells which can be extracted from the distribution of (**B**) for several different time points between 10 and 24 h (black to light gray). Data are fitted to dose-response functions (dashed lines). The ${\mathrm{EC}}_{50}$ values shift towards lower dose with increasing late time endpoints (black arrow); (**D**) The ${\mathrm{EC}}_{50}$ values are plotted in logarithmic scale against the endpoints. At an end-point of 18 h, the ${\mathrm{EC}}_{50}$ value approaches a constant value; (**E**, **F**) Time points $\tau_{50}$ (**E**) and a rate-dependent *σ* (**F**) for the cumulative distributions are plotted against dose. Both values exhibit dose-response behavior

The distribution of times of apoptosis provides a measure for the dynamic response of a cell population. We studied the evolution of the onset distribution with increasing NP dosage (from 0.1 μg · mL${}^{-1}$ to 100 μg · mL${}^{-1}$). In Figure 4A, normalized frequency distributions of onset times of the PI signal are plotted against time for various doses. It can be seen that with increasing NP dose, cell death onset shifts to earlier times. The same behavior is found in the corresponding time distribution for the pSIVA signal (data not shown), which also exhibits the same characteristic broadness in the distribution. The distributions are fitted by log-normal distributions. A log-normal distribution describes a process which is the product of many independent processes and is found (in our case as well as in literature) to fit the cell death distribution better than Gaussians [29]. For the lowest dose (0.1 μg · mL${}^{-1}$), the mean onset time is 27.8 ± 0.4 h, and for the highest dose it is shifted to the markedly earlier time of 8.9 ± 0.3 h. In addition, the distributions become narrower as the dosage is increased. The fraction of cells that dies is given by the cumulative sum. Figure 4B shows the cumulative sum against time and the fits to the integrated log-normal function. The time-course of the fraction of dead cells highlights the dependence of the dynamic response on dosage. At the highest dose almost all cells are dead after 24 h, whereas only 20% of those exposed to the lowest dose have died by this time. We evaluate this ”dynamic dose response” by plotting the time of 50% cell death, $\tau_{50}$, against dosage (Figure 4E). A drop in time-to-death with increasing dose can clearly be seen. The variance, *σ*, *i.e*., the width of the log-normal distribution, in contrast, increases with dose (Figure 4F). In order to retrieve standard ${\mathrm{EC}}_{50}$ values from the log-normal distributions shown in Figure 4A,B, we replotted the data as a whole as a function of dose rather than time (see Figure 4C). In this way, we obtain dose-response curves for various time points, *i.e*., effective end-points. We find that with increasingly late end-point the dose-response curves shift to lower dose values as depicted in Figure 4C. As a consequence, the ${\mathrm{EC}}_{50}$ value, *i.e*., the dose that result in 50% cell death, appears to depend on the time point of examination (Figure 4D). After a sufficiently long time, the ${\mathrm{EC}}_{50}$ values asymptotically approach a constant value. In our case, sufficiently long means 20 h, as is clearly seen in Figure 4D. However, it is not obvious *a priori* at which time point a reliable ${\mathrm{EC}}_{50}$ value can be obtained. In order to test if the heterogeneous response is caused by the NPs negative controls were performed (see Supplementary S4, Figure S3A,D). The negative control for the time-lapse measurement as well as a viability assay showed a high viability (95%) after four days. Further, in order to show the applicability of the single cell assay to other toxic agents, a dose response measurement for the anti-cancer drug staurosporine was performed (see Supplementary 4, Figure S3A–C).

### 2.3. Two-Parameter Correlation of Cell Death

**Figure 5:**
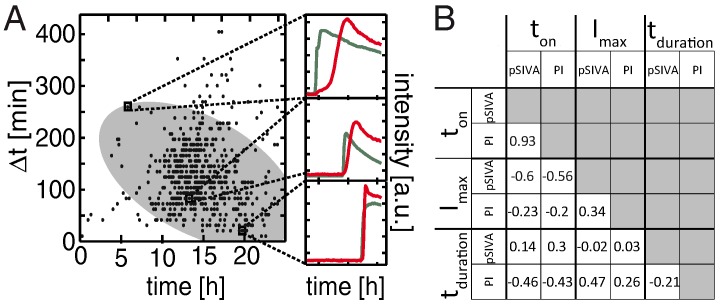
(**A**) The interval separating early-stage apoptosis (only pSIVA) from secondary necrosis (pSIVA+PI) varies depending on the onset time of apoptosis. This time difference Δt is plotted against the onset time. The grey ellipse represents a 2σ interval of a principal component analysis (PCA). Three representative time traces of apoptotic cells are depicted for better visualization: Cells that die at later time-points transit faster into secondary necrosis; (**B**) Different parameters of apoptotic cells are extracted from the time traces and displayed as a correlation matrix: onset times $t_{\mathrm{on}}$, maximum intensity $I_{\max}$ and duration of onset for pSIVA and PI. Maximal pSIVA intensity $I_{\max}$, and duration (spread) of onset time of PI $t_{\mathrm{duration}}$ are negatively correlated with the onset of apoptosis.

Next, the degree of correlation between onset times of the early and late apoptotic markers was examined. In Figure 5A, the interval Δt between the onset of the pSIVA signal $t_{\mathrm{pSIVA}}$ and that of the PI signal $t_{\mathrm{PI}}$ is plotted against the time of cell death, which we again define as $t_{\mathrm{PI}}$. The scatter plot shows that there is a tendency for Δt to decrease with time of death. In fact, cells that die earlier, *i.e*., in the time window between 5 and 10 h exhibit a delay time of about 100 to 300 min while late events of cell death show almost no delays. The correlation is underlined by a principal component analysis (PCA), which shows a tilted major axis, as indicated by the grey ellipse in the scatter plot. The correlation between pSIVA and PI onset times is also well brought out by the Pearson’s correlation coefficient (for details, see data analysis in Experimental Section). The Pearson’s correlation in this case is 0.96, and hence clearly indicates a strong correlation at the scale from zero (no correlation) to one (full correlation). Extending this concept to all measurable parameters of the apoptotic time trace we also investigated the cross-correlations of the onset time $t_{\mathrm{on}}$ with maximal intensity $I_{\max}$, and duration of onset $t_{\mathrm{duration}}$ (*i.e*., the time of increase from background to maximum signal for both the pSIVA and PI fluorescence). The correlation matrix is shown in Figure 5B for a dose of 10 μg · mL${}^{-1}$. Interestingly, the maximal intensity $I_{\max}$(pSIVA) correlates negatively with the onset of apoptosis (−0.6/−0.56). This possibly indicates a connection with cell size or caspase activity, or it may be related to fluorescence bleaching. Indeed, the heterogeneity of the pSIVA fluorescence intensity depends on the amount of PhS exposed, and hence depends on the level of activation of caspase3 [30,31]. Also the maximal intensity $I_{\max}$ (PI) is negatively correlated, albeit weakly, with the onset of apoptosis $t_{\mathrm{on}}$ (−0.23, −0.2). Likewise $I_{\max}$ (PI) is weakly correlated with $I_{\max}$(pSIVA) (0.34). Both pSIVA and PI signals depend on cell size, which is in turn cell-cycle dependent. However, there is also substantial intrinsic cell-to-cell variation in the size of the nucleus (see Supplementary Figure S2B) [32,33]. For completeness, we also show the correlation of the duration times of the onset. The duration $t_{\mathrm{duration}}$(PI) is negatively correlated with the onset of apoptosis $t_{\mathrm{on}}$ (−0.46/−0.43). $t_{\mathrm{duration}}$(PI) moderately correlates with $I_{\max}$ of pSIVA (0.47). Cells that die late undergo more rapid loss of membrane integrity, which leads to a faster increase in the PI signal. pSIVA $t_{\mathrm{duration}}$ does not, or only weakly, correlates with other parameters (0.14, 0.3, −0.02, and 0.03).

## 3. Discussion

In this work, we demonstrate the advantages of time-resolved analysis of single cell arrays on microstructured substrates for studies of the dynamics of NP-induced cell death. The individual cell traces permit virtually continuous monitoring of apoptotic events indicated by the markers pSIVA and PI. The wealth of information provided by this approach, in comparison to standard population-based toxicity tests, is obvious. The technique has allowed us to demonstrate that the time distribution function of apoptosis onset is dose dependent, and to introduce the $\tau_{50}$ value as a measure for fast or slow toxic efficacy. Conversely, the ${\mathrm{EC}}_{50}$ value is seen to depend on the time-point of measurement. The dependence of the ${\mathrm{EC}}_{50}$ value has been discussed previously [34]. It has also been noted that a time-dependent ${\mathrm{EC}}_{50}$ becomes exceedingly problematic in the low-dose regime, when toxicity occurs on time scales that exceed the duration of experimental observations [35,36]. In this case it can be argued that the $\tau_{50}$ value is the more useful measure. In the conventional approach, the evaluation of an ${\mathrm{EC}}_{50}$ value for weakly toxic nanomaterials is complicated not only by the choice of end-point, but also involves the risk of under- or over-estimating the toxic effect. In contrast to highly time-resolved measurements of many cells, our methodology allows us to extrapolate from the behavior of the relevant measured curve in order to get a first indication of toxicity risk in *in-vitro* systems.

A second major advantage of single cell data with high time resolution lies in the fact that successive events can be correlated in time. As described earlier, single-cell time-courses yield distinct onset times showing that the pSIVA signal precedes the PI signal. It is generally accepted that a difference in onset time between the two events is indicative of apoptosis-induced cell death. Hence in our case, as we clearly observe a pSIVA-PI time difference — at least in the early phase of NP exposure — cells death is induced via the apoptosis pathway. However, as the experiment proceeds, the delay between the onset of pSIVA and PI activation becomes shorter. Short delay times are interpreted as a signature of necrotic or necroptotic cell death. In our set-up, the number of necrotic cells rises with increasing duration of the experiment. This time-dependent upsurge in the incidence of necrosis in toxicity assays has been reported before, and has two possible causes. First, failure to eliminate apoptotic bodies, due to the lack of phagocytes *in-vitro*, promotes the transition from apoptotic to necrotic cell death [22,23]. Secondly, cells that undergo apoptosis affect neighboring cells towards cell death [37]. In addition, accumulation of NPs in cells over time, with a concomitant decrease in cell fitness, might contribute the increase of necrosis. At all events, the time-resolved single-cell approach quantifies the necrotic shift in the cell culture death with unprecedented directness. The utility of the single-cell strategy for exploring temporal correlations between fluorescent signals can also be extended by employing multiple markers indicative for distinct cellular events. As shown by the work of Wang et al. multiple markers can be used to elucidate the pathway induced by NPs [21].

## 4. Experimental Section

### 4.1. Cell Culture

HuH7 cells were cultured in RPMI supplemented with 2 mM l-glutamine (c-c-pro) and 10% fetal calf serum. Cells were grown to 70%–80% confluence, trypsinized and centrifuged at 1000 rcf for 3 min. Cell pellets were re-suspended in either cell medium or, for the experiments in Leibovitz’s L15 medium with GlutaMAX (Gibco), 10% fetal calf serum and 1 mM calcium chloride. Cells were stained with polarity Sensor for Viability and Apoptosis (pSIVA-IANBD, Novus Biologicals) [38,39] (the stock solution was diluted 1:50) and 1 μM propidium iodide (Novus Biologicals). Cell dyes were added without any further washing step. As a positive control for cell death staurosporine (Sigma Aldrich) was used at doses between 0.5 and 25 μM.

### 4.2. NP Preparation and Characterization

Core size and shape of the $\mathrm{PS}-{\mathrm{NH}}_{2}$ NPs (provided by QualityNano, UCD) were determined with a transmission electron microscope (TEM). NPs were adsorbed onto a Formvar/carbon film-coated grid and were observed with a Jeol 1011 TEM. Sizes of the NPs were determined with ImageJ and further analyzed in MATLAB (see Supplementary 1 Figure S1). Further characterization was carried out in the context of the QualityNano project [10,40]. Prior to use, NPs were vortexed for 1 min to obtain maximal dispersity, although aggregates of >1 μm were still present. Cells were exposed either to $\mathrm{PS}-{\mathrm{NH}}_{2}$ (0.1 μg · mL${}^{-1}$, 1 μg · mL${}^{-1}$, 10 μg · mL${}^{-1}$, and 100 μg · mL${}^{-1}$, corresponding to a surface concentration of 14 ng · cm${}^{-2}$ to 14 μg · cm${}^{-2}$). NPs were kept in solution for the duration of the measurement.

### 4.3. Micropatterning

The micro-structured surfaces were produced by selective oxygen plasma treatment (Femto Diener, 40 W for 3 min) on a topas substrate (μ-dishes ibidi GmbH) with subsequent passivation using either μ-dishes or 8 well slides. Selectivity was achieved using a polydimethylsiloxane (PDMS) stamp (cast from a master produced by photolithography) as a mask. The parts exposed to plasma were passivated with 1 mg · mL${}^{-1}$ PLL(20k)-g(3.5)-PEG(2k) (SuSoS AG) in aqueous buffer (10 mM HEPES pH 7.4 and 150 mM NaCl). The remaining sectors were rendered cell adherent by exposure to 50 μg · mL${}^{-1}$ fibronectin (YoProteins) for 1 h. The samples were thoroughly rinsed with PBS and stored in cell medium at room temperature prior to seeding (8000 cells per 8 well and 50,000 per μ-dish).

### 4.4. Time-Resolved Fluorescence Microscopy

Images were taken with an inverted Nikon Ti Eclipse microscope with phase-contrast and a fluorescent lamp with multiple filter sets (GFP, propidium iodide). Samples were kept at a constant temperature of 37 ${}^{{}^{\circ}}$C with an ibidi heating system (ibidi GmbH). Pictures were recorded every 10 min over a period of between 24 and 48 h.

### 4.5. Image and Data Analysis

Raw images were pre-processed in ImageJ. For the image analysis on the cell grid and a background correction the in-house plug-in Microwell Analysis was used. Multiple occupied sites, determined at the end of the measurement, were filtered out. The time of onset of apoptosis/secondary necrosis was defined as the point at which the pSIVA/PI signal first exceed the threshold of 25% of the basal background . This definition was in good agreement with manual tracking. The duration of the onset time was determined as the interval between the first local maximum of the smoothed curve and the crossing of the threshold. The dose-response of the log-normal distribution of dead cells dc(t) was fitted by

$$dc\left(t\right)=A*exp{\left(\frac{log(t)-\mu }{\sqrt{\left(2\sigma \right)}}\right)}^{2}$$

and the fraction of dead cells F(t), the cumulative sum of the distribution, by

$$F\left(t\right)=0.5*\left[1+erf{\left(\frac{log(t)-\mu }{\sqrt{\left(2\sigma^{2}\right)}}\right)}^{2}\right]$$

where *A* denotes amplitude, *t* the time, *σ* the standard deviation, and μ the mean. The dose-response curves for the fraction of dead cells F(dose) in Figure 4C were fitted by

$$F\left(dose\right)=y_{0}+\frac{-y_{0}}{1+{\left(EC_{50}/dose\right)}^{n}}$$

with rate *n*, the basal rate $y_{0}$, and the ${\mathrm{EC}}_{50}$ value at which 50% of cells are dead. Onset times of the two markers were analyzed, and the subsequent correlation analysis was carried out, in MATLAB. For the principal component analysis, the eigenvectors were calculated for the data set of $t_{\mathrm{PI}}$ and Δt. In Figure 5A, the plotted axis of the ellipse correspond to those eigenvectors with lengths of two standard deviations *σ*. The ellipse is centered on the mean. The correlation coefficient was defined by Pearson’s correlation :

$$\rho \left(X,Y\right)=\frac{Cov(X,Y)}{\sigma (X)\sigma (Y)}$$

with the covariance Cov(X,Y) of the two parameters *X* and *Y* and their related standard deviation *σ*.

## 5. Conclusions

In summary, we provide the first kinetic analysis of NP-induced cell death based on single cell arrays. The resulting time-resolved measurements enable us to measure the onset distribution function of NP-induced apoptosis and a differentiated time-dependent picture of the NP dose-response relationship emerges. Future use of micro-arrays for high-throughput acquisition of single cell time-lapse data will pave the way to kinetic investigations of cell death in *in-vitro* and could potentially uncover aspects of the action NPs trapped within cells, thus allowing for the detailed characterization of nanomaterial pathways using single cell cross-correlation. Hence automated microscopy combined with micro-array technology might evolve into a potent platform for nanomaterial characterization and beneficially complement toxicity risk assessment.

## Supplementary Materials

The following are available online at http://www.mdpi.com/2076-3905/5/2/8/s1.

## References

[1] Colvin V.L. (2003). The potential environmental impact of engineered nanomaterials. Nat. Biotech..

[2] Nel A., Xia T., Madler L., Li N. (2006). Toxic potential of materials at the nanolevel. Science.

[3] Anguissola S., Garry D., Salvati A., O’Brien P.J., Dawson K.A. (2014). High content analysis provides mechanistic insights on the pathways of toxicity induced by amine-modified polystyrene nanoparticles. PLoS ONE.

[4] Elsaesser A., Howard C.V. (2012). Toxicology of nanoparticles. Adv. Drug Deliv. Rev..

[5] Love S.A., Maurer-Jones M.A., Thompson J.W., Lin Y.S., Haynes C.L. (2012). Assessing nanoparticle toxicity. Annu. Rev. Anal. Chem..

[6] Lu X., Liu Y., Kong X., Lobie P.E., Chen C., Zhu T. (2013). Nanotoxicity: A growing need for study in the endocrine system. Small.

[7] Fonseca C., Simões S., Gaspar R. (2002). Paclitaxel-loaded PLGA nanoparticles: preparation, physicochemical characterization and in vitro anti-tumoral activity. J. Control. Release.

[8] Kommareddy S., Amiji M. (2005). Preparation and evaluation of thiol-modified gelatin nanoparticles for intracellular DNA delivery in response to glutathione. Bioconjugate Chem..

[9] Jose G.P., Santra S., Mandal S.K., Sengupta T.K. (2011). Singlet oxygen mediated DNA degradation by copper nanoparticles: potential towards cytotoxic effect on cancer cells. J. Nanobiotechnology.

[10] Bexiga M.G., Varela J.A., Wang F., Fenaroli F., Salvati A., Lynch I., Simpson J.C., Dawson K.A. (2011). Cationic nanoparticles induce caspase 3-, 7- and 9-mediated cytotoxicity in a human astrocytoma cell line. Nanotoxicology.

[11] Wlodkowic D., Skommer J., Darzynkiewicz Z. (2009). Flow cytometry-based apoptosis detection. Methods Mol. Biol..

[12] Summers H.D., Rees P., Holton M.D., Rowan Brown M., Chappell S.C., Smith P.J., Errington R.J. (2011). Statistical analysis of nanoparticle dosing in a dynamic cellular system. Nat. Nano.

[13] Xia X., Owen M.S., Lee R.E., Gaudet S. (2014). Cell-to-cell variability in cell death: can systems biology help us make sense of it all?. Cell Death Differ..

[14] Spencer S.L., Gaudet S., Albeck J.G., Burke J.M., Sorger P.K. (2009). Non-genetic origins of cell-to-cell variability in TRAIL-induced apoptosis. Nature.

[15] Love K.R., Bagh S., Choi J., Love J.C. (2013). Microtools for single-cell analysis in biopharmaceutical development and manufacturing. Trends Biotechnol..

[16] Ware M.J., Godin B., Singh N., Majithia R., Shamsudeen S., Serda R.E., Meissner K.E., Rees P., Summers H.D. (2014). Analysis of the Influence of Cell Heterogeneity on Nanoparticle Dose Response. ACS Nano.

[17] Aftab O., Nazir M., Fryknas M., Hammerling U., Larsson R., Gustafsson M.G. (2014). Label free high throughput screening for apoptosis inducing chemicals using time-lapse microscopy signal processing. Apoptosis.

[18] Albeck J.G., Burke J.M., Aldridge B.B., Zhang M., Lauffenburger D.A., Sorger P.K. (2008). Quantitative analysis of pathways controlling extrinsic apoptosis in single cells. Mol. Cell.

[19] Forrester H.B., Albright N., Ling C.C., Dewey W.C. (2000). Computerized video time-lapse analysis of apoptosis of REC:Myc cells X-irradiated in different phases of the cell cycle. Radiat. Res..

[20] Milani S., Baldelli Bombelli F., Pitek A.S., Dawson K.A., Rädler J. (2012). Reversible *versus* irreversible binding of transferrin to polystyrene nanoparticles: Soft and hard corona. ACS Nano.

[21] Wang F., Yu L., Monopoli M.P., Sandin P., Mahon E., Salvati A., Dawson K.A. (2013). The biomolecular corona is retained during nanoparticle uptake and protects the cells from the damage induced by cationic nanoparticles until degraded in the lysosomes. Nanomedicine.

[22] Silva M.T. (2010). Secondary necrosis: The natural outcome of the complete apoptotic program. FEBS Lett..

[23] Vanden Berghe T., Grootjans S., Goossens V., Dondelinger Y., Krysko D.V., Takahashi N., Vandenabeele P. (2013). Determination of apoptotic and necrotic cell death in vitro and in vivo. Methods.

[24] Wlodkowic D., Faley S., Zagnoni M., Wikswo J.P., Cooper J.M. (2009). Microfluidic single-cell array cytometry for the analysis of tumor apoptosis. Anal. Chem..

[25] Röttgermann P.J., Hertrich S., Berts I., Albert M., Segerer F.J., Moulin J.F., Nickel B., Rädler J.O. (2014). Cell motility on polyethylene glycol block copolymers correlates to fibronectin surface adsorption. Macromol. Biosci..

[26] Röttgermann P.J.F., Piera Alberola A., Rädler J.O. (2014). Cellular Self-Organization on Micro-Structured Surfaces. Soft Matter.

[27] Waring M.J. (1965). Complex formation between ethidium bromide and nucleic acids. J. Mol. Biol..

[28] Kim J.A., Åberg C., de Cárcer G., Malumbres M., Salvati A., Dawson K.A. (2013). Low dose of amino-modified nanoparticles induces cell cycle arrest. ACS Nano.

[29] Furusawa C., Suzuki T., Kashiwagi A., Yomo T., Kaneko K. (2005). Ubiquity of log-normal distributions in intra-cellular reaction dynamics. Biophysics.

[30] Frasch S.C., Henson P.M., Kailey J.M., Richter D.A., Janes M.S., Fadok V.A., Bratton D.L. (2000). Regulation of phospholipid scramblase activity during apoptosis and cell activation by protein kinase C*δ*. J. Biol. Chem..

[31] Lee S.H., Meng X.W., Flatten K.S., Loegering D.A., Kaufmann S.H. (2013). Phosphatidylserine exposure during apoptosis reflects bidirectional trafficking between plasma membrane and cytoplasm. Cell Death Differ..

[32] Edens L.J., White K.H., Jevtic P., Li X., Levy D.L. (2013). Nuclear size regulation: from single cells to development and disease. Trends Cell Biol..

[33] Huber M.D., Gerace L. (2007). The size-wise nucleus: Nuclear volume control in eukaryotes. J. Cell Biol..

[34] Krippendorff B.F., Neuhaus R., Lienau P., Reichel A., Huisinga W. (2009). Mechanism-based inhibition: Deriving K(I) and k(inact) directly from time-dependent IC(50) values. J. Biomol. Screen.

[35] Vandenberg L.N., Colborn T., Hayes T.B., Heindel J.J., Jacobs D.R.J., Lee D.H., Shioda T., Soto A.M., vom Saal F.S., Welshons W.V. (2012). Hormones and endocrine-disrupting chemicals: Low-dose effects and nonmonotonic dose responses. Endocr Rev..

[36] Yedjou C., Moore P., Tchounwou P. (2006). Dose- and time-dependent response of human leukemia (HL-60) cells to arsenic trioxide treatment. Int. J. Environ. Res. Public Health.

[37] Gregory C.D., Pound J.D., Devitt A., Wilson-Jones M., Ray P., Murray R.J. (2009). Inhibitory effects of persistent apoptotic cells on monoclonal antibody production in vitro: Simple removal of non-viable cells improves antibody productivity by hybridoma cells in culture. mAbs.

[38] Kim Y.E., Chen J., Chan J.R., Langen R. (2010). Engineering a polarity-sensitive biosensor for time-lapse imaging of apoptotic processes and degeneration. Nat. Meth..

[39] Kim Y.E., Chen J., Langen R., Chan J.R. (2010). Monitoring apoptosis and neuronal degeneration by real-time detection of phosphatidylserine externalization using a polarity-sensitive indicator of viability and apoptosis. Nat. Protoc..

[40] Wang F., Bexiga M.G., Anguissola S., Boya P., Simpson J.C., Salvati A., Dawson K.A. (2013). Time resolved study of cell death mechanisms induced by amine-modified polystyrene nanoparticles. Nanoscale.

