# Supplementary material for: Time-Resolved Study of Nanoparticle Induced Apoptosis Using Microfabricated Single Cell Arrays
Source: Microarrays (Basel). 2016 Apr 15;5(2):8. doi: 10.3390/microarrays5020008 (PMC5003484; doi:10.3390/microarrays5020008)
Supplement: Supplementary file 1 [file microarrays-05-00008-s001.zip › microarrays-05-00008- SI.pdf]

Supplementary Materials: Time-Resolved Study of Nanoparticle Induced Apoptosis Using Microfabricated Single Cell Arrays

Peter Johan Friedrich Röttgermann[1], Kenneth Adrian Dawson [2] and Joachim Oskar Rädler [1,*]

Supplementary Material

Supplementary 1

Core size and shape of the amine PS-NPs were characterized with TEM. NPs were adsorbed onto a Formvar/carbon film-coated grid and were observed with a Jeol 1011 TEM. The mean size of the particles was determined to be 35 μm (Figure S1C). Note that aggregated particles were observed in the experiment as well as in TEM images (Figure S1B).

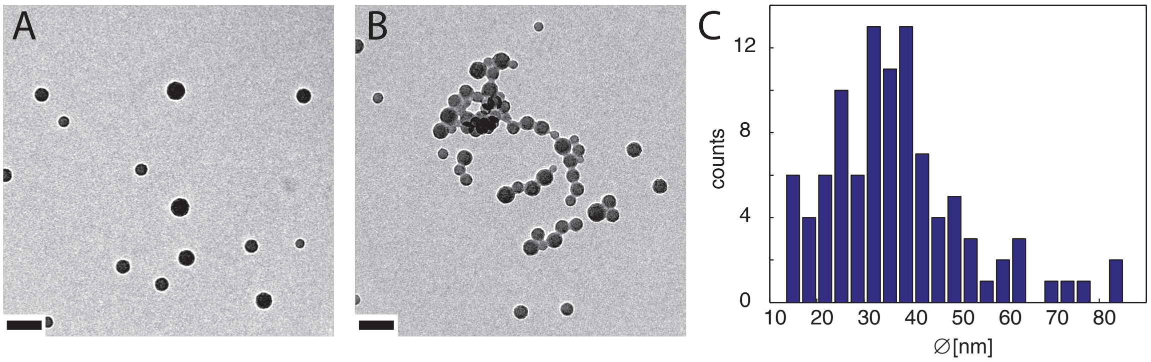

Figure S1. TEM images of monodispersed (**A**) and agglomerated (**B**) $PS - NH_2$ particles. Scale Bar: 100 μm. (**C**) Size distributions of the NPs with mean diameter particle size 35 μm.

Supplementary 2

Time-lapse movie at 30 h of cells on a single-cell array. Cells are stained with pSIVA-IANBD, which fluoresces green upon binding to phosphatidylserine, and with propidium iodide, which fluoresces red when bound to nuclear DNA upon loss of plasma membrane integrity. After 8 h the first cells undergo cell death. The heterogeneity in time of onset of apoptosis can be clearly seen.

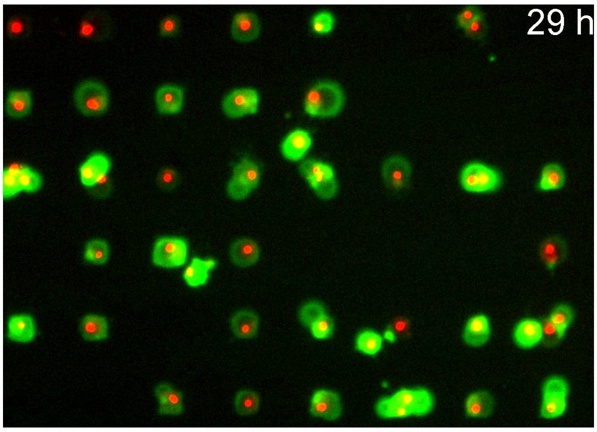

Supplementary 3

The maximal signal intensity attained differs between single cells. Figure S2 shows the frequency distributions for the pSIVA signal (corrected for bleaching) and the PI signal for an NP dosage of 10 $\mu g \cdot mL^{-1}$. The plots are approximately Gaussian distributed. The differences in the maximal pSIVA signal can be explained by variation in the amount of exposed PS and by cell size, the differences in the PI signal by cell-cycle phase and the size distribution of the cell nuclei. The peak around zero is attributed to the healthy cells, which exhibit a low basal level of fluorescence.

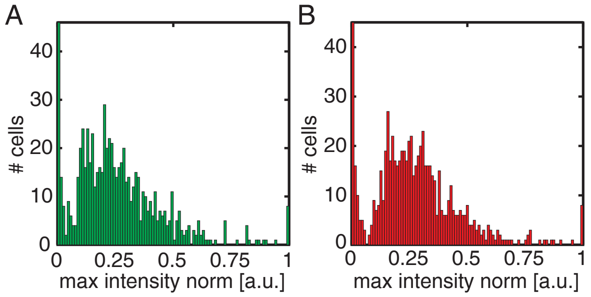

Figure S2. Frequency distribution of normalized maximum intensity for (**A**) pSIVA and (**B**) PI signal for a dose of 10 $\mu g \cdot mL^{-1}$. Signals are corrected for bleaching.

Supplementary 4

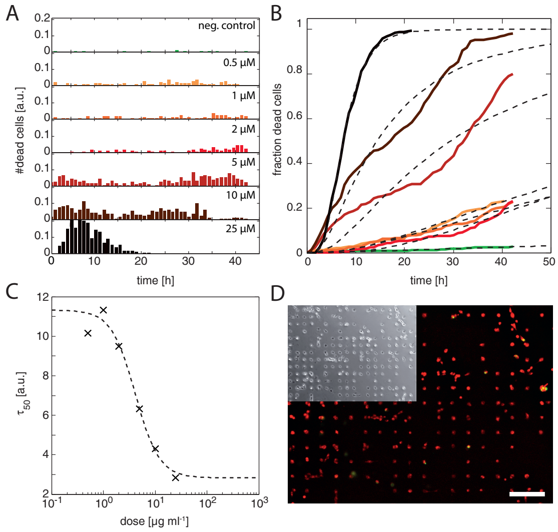

Figure S3. (**A**) Frequency distribution of dead cells for negative control (no treatment) and for STS at various doses (0.5 to 25 μM). A bimodal cell distribution can be determined. Doses higher than 5 μm exhibit a second population of early cell death in the first 20 h. (**B**) Cumulative fraction of dead cells in relation to time are plotted for the negative control and STS. Regardless the bimodality, the cell distributions are fitted to log-normal functions. (**C**) Graph with time points τ_{50} for the cumulative distributions in dependence of dose. For STS, a dose-response behavior is observed. (**D**) Exemplary fluorescent image and brightfield image as inset of a viability test for the negative control (no addition of NPs and cell death markers). Red indicates viable cells (mitochondrial activity) and green compromised cell membranes (nucleus staining). Scale bar: 200 μm.

In order to show that the toxicity studies are not only limited to NPs we performed time-lapse measurements in which cells were treated with the anti-cancer drug staurosporine (STS) at doses of 0.5, 1, 2, 5, 10, and 25 μM. Figure S3 A show the cell death distributions for the various doses as well as for a negative control (with no STS or NP) over a period of 43 h. Depending on the dose, a bimodal distribution of cell death can be distinguished. For a dose regime of 0.5 to 2 μM cells die toward late time points, whereas for 5 and 10 μM two fraction of cell death can be distinguished: a fraction which die in the first 20 h and a fraction which induce cell death around 30 h. At the highest dose (25 μM), all cells die in the first 20 h. The fraction of cell death is plotted against time in Figure S3 B. The toxicity level for the negative control is with only 5% low. The cumulative distribution are fitted to log-normal functions neglecting the bimodal behavior at higher doses (see Figure S3 C). For STS, a time-dependent dose-response behavior is observed as also seen for the amino-modified PS NPs.

An additional test was performed to test if cells stay viable on the cell array over a period of 4 days. A live-dead assay with c12-resazurin and SYTOX green was used (see Figure S3D) which measures the metabolic activity as well as the integrity of the plasma membrane. A red color indicate a healthy metabolism whereas a green color (stained nucleus) indicate a compromised cell membrane and hence dead cells. After four days a high viability of 95% $\pm$ 0.6 % was observed showing that the micropattern has no adverse effect on the cells.
